# Gastroesophageal reflux in congenital diaphragmatic hernia survivors: objective diagnostics reveal clinically relevant disease beyond reported symptoms

**DOI:** 10.1007/s00383-026-06409-3

**Published:** 2026-05-19

**Authors:** Julia Elrod, Katharina Klensch, Rasul Khasanov, Meike Weis, Michaela Klinke, Caroline Riemer, Michael Boettcher, Richard Martel

**Affiliations:** 1 Department of Pediatric Surgery, University Medical Center Mannheim, Medical Faculty Mannheim, Heidelberg University, Theodor-Kutzer-Ufer 1-3, 68167 Mannheim, Germany; 2 Department of Clinical Radiology and Nuclear Medicine, University Medical Center Mannheim, Medical Faculty Mannheim, Heidelberg University, Mannheim, Germany

**Keywords:** Congenital diaphragmatic hernia, Gastroesophageal reflux, Prevalence, Thriving, Lung function

## Abstract

**Purpose:**

While gastroesophageal reflux disease (GERD) is a frequent comorbidity in congenital diaphragmatic hernia (CDH), there is no consensus about a standardized screening strategy within the speciality. Reported prevalence is heterogeneous, and the role of anti-reflux surgery remains controversial. This single-center study aimed to assess the reliability of symptom inquiry, age-dependent prevalence, the impact of GERD on thriving and lung function, and CDH-inherent predispositions.

**Methods:**

Records of 1250 CDH-patients treated between 2000 and 2022 were reviewed. Associations of GER with symptoms, weight gain, and respiratory function were analyzed. A multivariate regression model was used to identify risk factors.

**Results:**

Of 1250 patients, 916/964 survivors were followed for a median of 8.7 years [IQR 4.2–13.4]. Reflux-associated symptoms were reported in 18.0%. Esophagogastroduodenoscopy (EGD) was performed in 85 patients, pH-metry in 58 (50 pH-MII). Pathologic findings were detected in 5.2% of the patients. 115 contrast studies showed reflux in further 8.2%. Anti-reflux surgery was performed in 6.9%. Reported gastrointestinal symptoms were associated with higher 24 h-reflux count (74.9 ± 40.7 vs. 51.0 ± 32.8, *p* = 0.033). Weight gain correlated negatively with esophagitis severity (*r*=-0.25, *p* = 0.04) and acid exposure time (-0.41, *p* = 0.004). Obstructive lung function correlated with the endoscopic grade of esophagitis (*r*=-0.52, *p* = 0.04). Larger CDH-defect size and liver herniation were the only significant predisposing factors for GERD.

**Conclusion:**

CDH patients, especially those with large defects, are at high risk for GERD, which impairs thriving and lung function. Because symptoms poorly reflect objective findings, structured follow-up including EGD and pH-MII is essential.

**Supplementary Information:**

The online version contains supplementary material available at 10.1007/s00383-026-06409-3.

## Introduction

Gastroesophageal reflux (GER) is a frequent comorbidity in survivors of Congenital Diaphragmatic Hernia (CDH) [34]. While the exact pathophysiologic mechanisms remain unclear, it is assumed that changes in the lower esophageal sphincter (LES), weakening of the diaphragmatic crura, and increased abdominal pressure following surgical repair contribute to its development [20].

Untreated GER can cause feeding difficulties and symptoms such as heartburn and vomiting, thereby impairing quality of life. Persistent GERD is a major risk factor for mucosal changes, including Barrett esophagus and adenocarcinoma, and may lead to failure to thrive [5]. This pathologic form of GER is referred to as gastroesophageal reflux disease (GERD) [29].

For this reason, early identification and appropriate treatment of GERD are crucial. Since it presents with heterogeneous symptoms, especially in younger children [5], diagnostic verification is required. For this purpose, 24-hour pH-metry with multichannel intraluminal impedance (24-hour pH-MII) and esophagogastroduodenoscopy (EGD) with or without biopsies are available.

To date, no standardized screening strategy for GERD in CDH survivors has been established [21, 22] and no universal diagnostic principles for GERD in children exist [5]. In adults, GERD diagnosis is based on symptoms, abnormal reflux index in 24-hour pH-MII, and presence of esophagitis graded by Los Angeles Classification in EGD [32].

The aim of this single-center study in CDH survivors was to evaluate the dependability of symptom inquiry, age-dependent prevalence of GER, its potential impact on thriving and lung function, and to identify CDH-inherent predisposing factors.

## Methods

### Patient cohort

The electronic patient records of 1,250 patients treated for CDH at the Department of Pediatric Surgery of the University Medical Center Mannheim were analyzed. All data were prospectively collected within the Mannheim CDH follow-up study. The patients were born between 2000 and 2022, with the majority delivered at our hospital. Two patients with synchronous esophageal atresia (EA) in addition to CDH were excluded from analysis. Initial surgical defect closure was performed either by thoracoscopy or laparotomy. Laparotomy was preferred in patients with persistent pulmonary hypertension, i.e., requiring extracorporeal membrane oxygenation (ECMO). Primary closure was performed whenever possible without tension on the diaphragm, otherwise a Goretex patch was used.

Demographic data, including gestational age, sex at birth, side and size of the hernia according to the CDH Study Group (CDH-SG) [9], type and surgical access of hernia repair, need for ECMO and liver herniation into the thoracic cavity (liver-up) were collected.

### Follow-up program

All CDH survivors managed actively at our institution are invited at the age of 0.5, 1, 2, 4 and 6 years, thereafter the schedule followed either 8, 12, and 16 years (earlier protocol) or 10, 14, and 18 years (structured follow-up program). In case of complaints, patients were examined at any time point and in case of surgery due to recurrence, the initial postoperative follow up included visits 0.5, 1, 2 years after the operation. Body weight and length were measured at each visit, and z-scores were calculated according to Kromeyer-Hauschild et al. [11]. At each visit, symptoms of GER were assessed and in case of alarm signs, further diagnostic work-up was performed, including EGD and 24-hour pH-MII. Alarm signs were summarized for statistical analysis into the categories feeding difficulties, abdominal complaints, respiratory symptoms.

### Reflux diagnostics

Whenever EGD was performed, endoscopic findings, use of proton pump inhibitors (PPI) prior to EGD, longitudinal growth data, biopsy and pH-MII results and information on anti-reflux surgery (ARS) were documented.

Esophagitis observed during EGD was classified according to the Los Angeles (LA) classification [1]. Histological esophagitis was categorized as mild, florid or ulcerative based on the pathologist’s report. Health records were also screened for Barrett’s esophagus. The analyzed output of the 24-hour pH-MII was acid exposure time (AET) and reflux count. With unequivocal endoscopic esophagitis, pH-MII was not performed.

Contrast studies to evaluate the anatomy of the gastroesophageal junction and directly detect reflux were performed with iso-osmolar iodine-based contrast agent at a total maximum volume of 5 ml/kg contrast agent diluted 1:1 with water or juice. The passage into the stomach was monitored by fluoroscopy, intermittent pictures were taken during the first 5 min after complete esophagogastric passage. Intermittent pictures were taken supplemented by turning to supine position or in 30° right sided position accompanied by drinking of water (siphon test).

To dichotomize the data for statistical analysis, the following diagnostic findings were defined as pathologic: esophagitis grade ≥ A according to the Los Angeles classification (EGD); any grade of esophagitis in the biopsy; an AET ≥ 6% or reflux count 80/24 h in the pH-MII according to the Lyon Consensus 2.0 [8]; detection of pronounced reflux in upper gastrointestinal contrast study.

### Lung function

According to the current consensus recommendations from the European Respiratory Society (ERS) and American Thoracic Society (ATS), the following criteria were used to determine whether a maximal effort was achieved and acceptable measurements were obtained: an immediate start of forced expiration to ensure that the forced expiratory volume in one second (FEV1) results from a maximal effort, a sharp rise in forced expiratory flow until peak flow was reached, and a complete exhalation to ensure attainment of a true forced vital capacity (FVC) [6]. Measurements that did not meet one of these criteria were excluded from the analysis.

The FVC was used to quantify potential restrictive ventilatory defects. The FEV1/FVC ratio, also known as the Tiffeneau Index [25], was used to quantify potential obstructive ventilatory impairment. The acquired spirometry values were analyzed as percent of the predicted values with respect to body measurements and age (%FVC and %FEV1/FVC).

### Statistics

Statistical analysis was performed using Statistical Analysis Software version 9.4 (SAS Institute Inc., Cary, North Carolina USA). Associations between categorical variables were assessed using contingency tables and tested for significance with the Chi-square or Fisher’s exact test. Continuous variables were reported as absolute values, arithmetic mean and standard deviations. Differences in non-normally distributed variables were assessed using the Mann-Whitney U-test with normal approximation and one-way ANOVA and analysis of covariance (ANCOVA) was used to adjust for potential confounders. Associations between ordinal variables were evaluated by the Spearman rank correlation and the Pearson’s correlation for continuous variables. Factors associated with reflux findings were analyzed by a multinomial logistic regression model calculating regression coefficients by the maximum likelihood method. Results from any analysis in this study were considered statistically significant if the p-value was below 0.05.

## Results

**Fig. 1:**
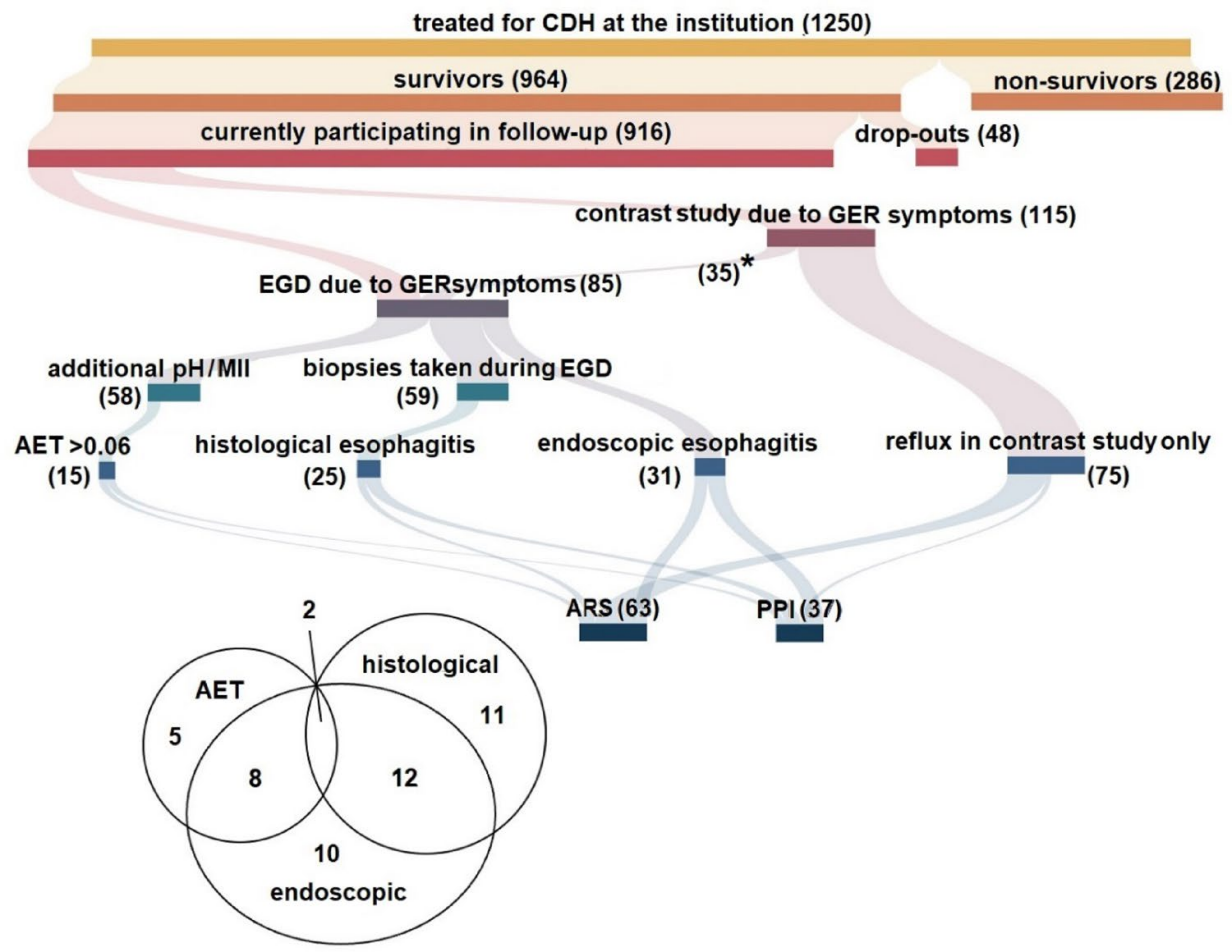
Flow chart showing the process of identification and therapy of reflux-patients within the CDH follow-up program. Bar lengths are proportionate to patient numbers. 85 patients were examined by EGD, histology, and/or pH-MII. Pathologic findings were present in 48/916 (5.2%) patients (exclusively EGD: *N* = 10; exclusively histology: *N* = 11; exclusively pH-MII: *N* = 5; combined EGD+histology: *N* = 12; combined EGD + pH-MII: *N* = 8; combined EGD+histology + pH-MII: *N* = 2). The total of 48 respects overlapping findings as specified in the Venn diagram. 115 patients were examined by contrast studies, 35 of these received subsequent EGD+histology + pH-MII (*), 13 of these had pathologic findings. In 75/80 further contrast studies, pronounced reflux was detected. Anti-reflux surgery was performed in 20 patients following EGD ± pH-MII and in 43 patients following contrast studies (total 63/916; 6.9%). *AET* acid exposure time, *ARS* anti-reflux surgery, *EGD* esophagogastroduodenoscopy, *pH-MII* 24-hour pH-metry with multichannel intraluminal impedance, *PPI* proton pump inhibitor, *initial contrast study, subsequent EGD+histology + pH-MII

**Fig. 2:**
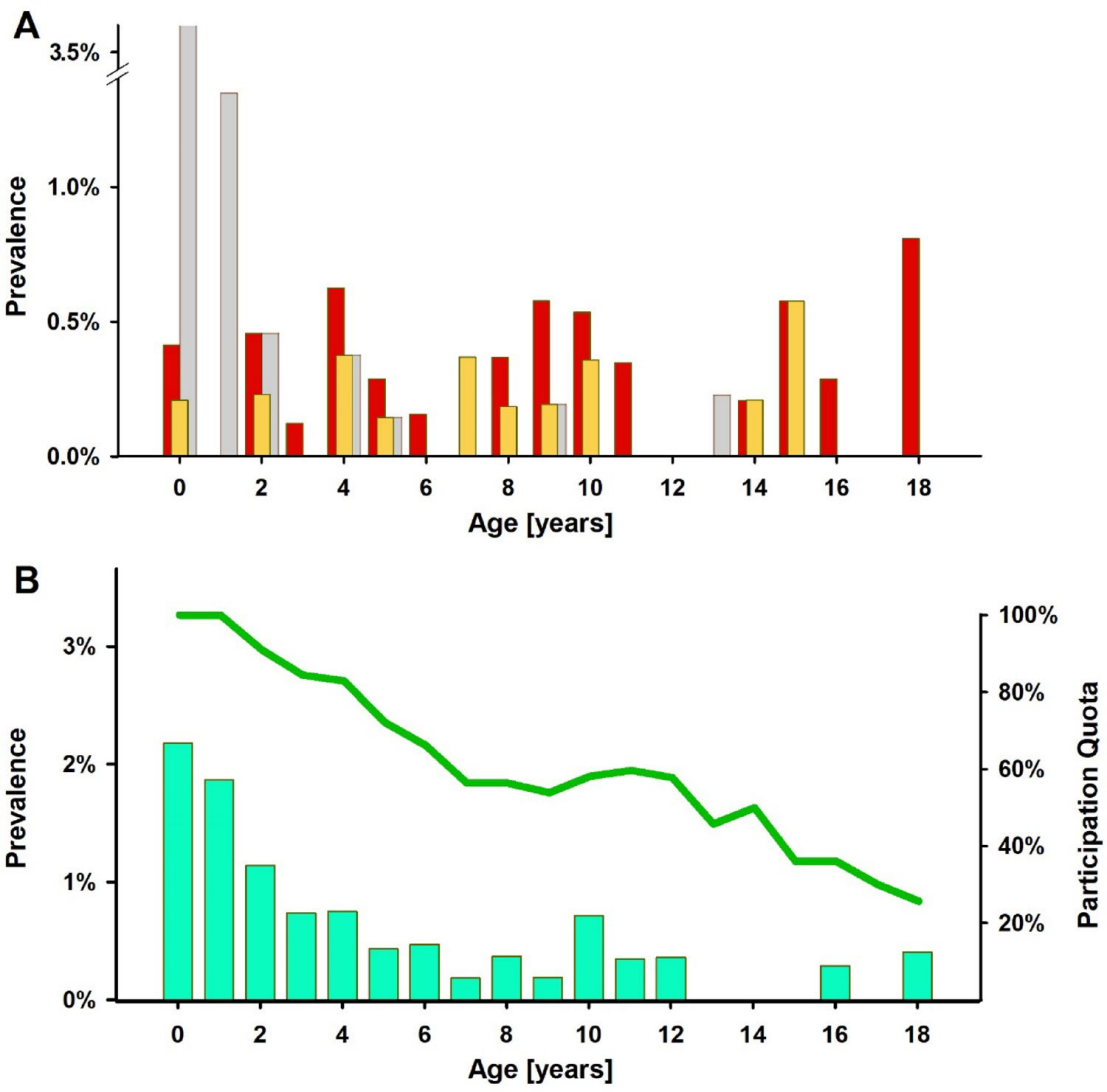
Age specific prevalence of reflux findings and anti-reflux surgery adjusted for the adherence to the follow-up program. Macroscopic esophagitis (red columns) and pathologic acid exposure time (yellow columns) was detected at all age levels comprising age-gaps that partially mirror the follow-up intervals (6, 10, 14 years) (**A**). Considering the decreasing participation of initially 964 CDH survivors (**B**, green line) it may be underestimated. Historically, reflux was frequently depicted in contrast radiographies in infants (grey columns, **A**). This was the age, at which most of the surgical interventions were performed (green columns, **B**)

1250 CDH-patients were treated at University Medical Center Mannheim between 2000 and 2022. 916 of the 964 survivors (95%) took part in the follow-up program (Fig. 1) with decreasing participation quota over time (Fig. 2b). Median follow up time was 8.7 years [IQR 4.2–13.4]. Details on disease severity and the initial course are shown in Supplement 1. 165 (18.0%) of these 916 patients (or caregivers) answered positive to the inquiry of reflux-associated symptoms (Fig. 1). EGD with biopsies were performed in 85 patients with additional 24-hour pH-metry in 58 patients (50 pH-MII).

The grade of endoscopic esophagitis was significantly correlated with Acid Exposure Time (AET) (ρ = 0.29, *p* = 0.04) and reflux count (*r* = 0.39, *p* = 0.01). An exclusive contrast study was performed in 80 patients, mainly in infants (Fig. 2a). 35 patients were first examined by contrast radiography while EGD was performed in a later visit.

### Diagnostic yield and distribution of pathological reflux findings

48 (5.2%) patients had pathologic findings in the EGD with optional pH-MII, two of them in all three modalities (macroscopic, histologic and in pH-MII), none of them had solely histologic pathologies combined with pathologic pH-MII; details are specified in Supplement 2. No Barrett esophagus was found. 88 (9.6%) of the followed-up survivors had reflux in the contrast study that was classified pathologic. 13 of them had pathologic findings in a later EGD (1.4%). In total, 123 (13.4%) were diagnosed with pathologic findings. Grades of pathologic findings and numbers of affected patients are specified in Supplement 2. Twenty (2.2%) patients with pathologic EGD and/or pH-MII-findings received anti-reflux surgery, 43 (4.7%) following contrast studies (Fig. 1).

### Symptom–diagnostic concordance

Within the 165 technically examined survivors, no significant association was detected between assessed symptoms including previous PPI-intake and presence of histologic esophagitis, endoscopic grade of esophagitis or reflux in the contrast radiography. Concerning pH-MII measures, abdominal complaints were significantly associated with higher reflux count (74.9 ± 40.7, 51.0 ± 32.8, *p* = 0.033, Table 1). Of note, the symptom categories are not mutually exclusive and were analyzed independently; a multivariate adjustment was not feasible given the limited sample size.

**Table 1:** Clinical symptoms as poor predictors of objective reflux

	Symptom	Ratio symptomatic/asymptomatic	Mean ± SD symptomatic	Mean ± SD asymptomatic	*p*-value (U-Test)
Acid Exp. Time [%]	Feeding difficulties	15/36	4.35 ± 6.31	6.21 ± 7.06	0.153
	Abdominal complaints	36/15	4.72 ± 5.46	7.93 ± 9.21	*0.410*
	Respiratory symptoms	15/36	4.53 ± 6.14	6.13 ± 7.14	*0.176*
	PPI-intake	40/11	5.53 ± 6.33	6.16 ± 8.78	*0.241*
24h-Reflux Count	Feeding difficulties	14/30	60.9 ± 49.2	71.9 ± 35.0	*0.124*
	Abdominal complaints	32/12	74.9 ± 40.7	51.0 ± 32.8	***0.033***
	Respiratory symptoms	13/31	63.1 ± 46.4	70.6 ± 37.3	*0.213*
	PPI-intake	37/7	71.3 ± 39.3	52.9 ± 42.1	0.218

Only abdominal complaints were significantly associated with higher reflux counts. Symptom categories with very small sample sizes (failure to thrive, regurgitation; *N* ≤ 7) were excluded due to insufficient statistical power. Note that symptom categories are not mutually exclusive

*Acid Exp Time* acid exposure time, *PPI* proton pump inhibitors, p was calculated using Mann–Whitney-U-Test

### Age-specific prevalence

In the present cohort, the prevalence of GERD substantiated by EGD with optional pH-MII was 5.2% while in 8.2% of the patients, a lone-standing contrast study showed reflux considered as pathologic. The age-related distribution (Fig. 2a) shows two gaps reflecting the standardized follow-up intervals. In addition to these, reassessments were appointed in case of complaints. The frequency of anti-reflux operations is consistent (Fig. 2b) with the number of pathologic findings (Fig. 2a). In patients from approximately 4 years of age, this is esophagitis and pathologic AET. Most of the operations per year of age have been performed at the age of 0–2 years, where the diagnosis was made mainly by contrast studies.

### Association between reflux metrics and growth

**Fig. 3:**
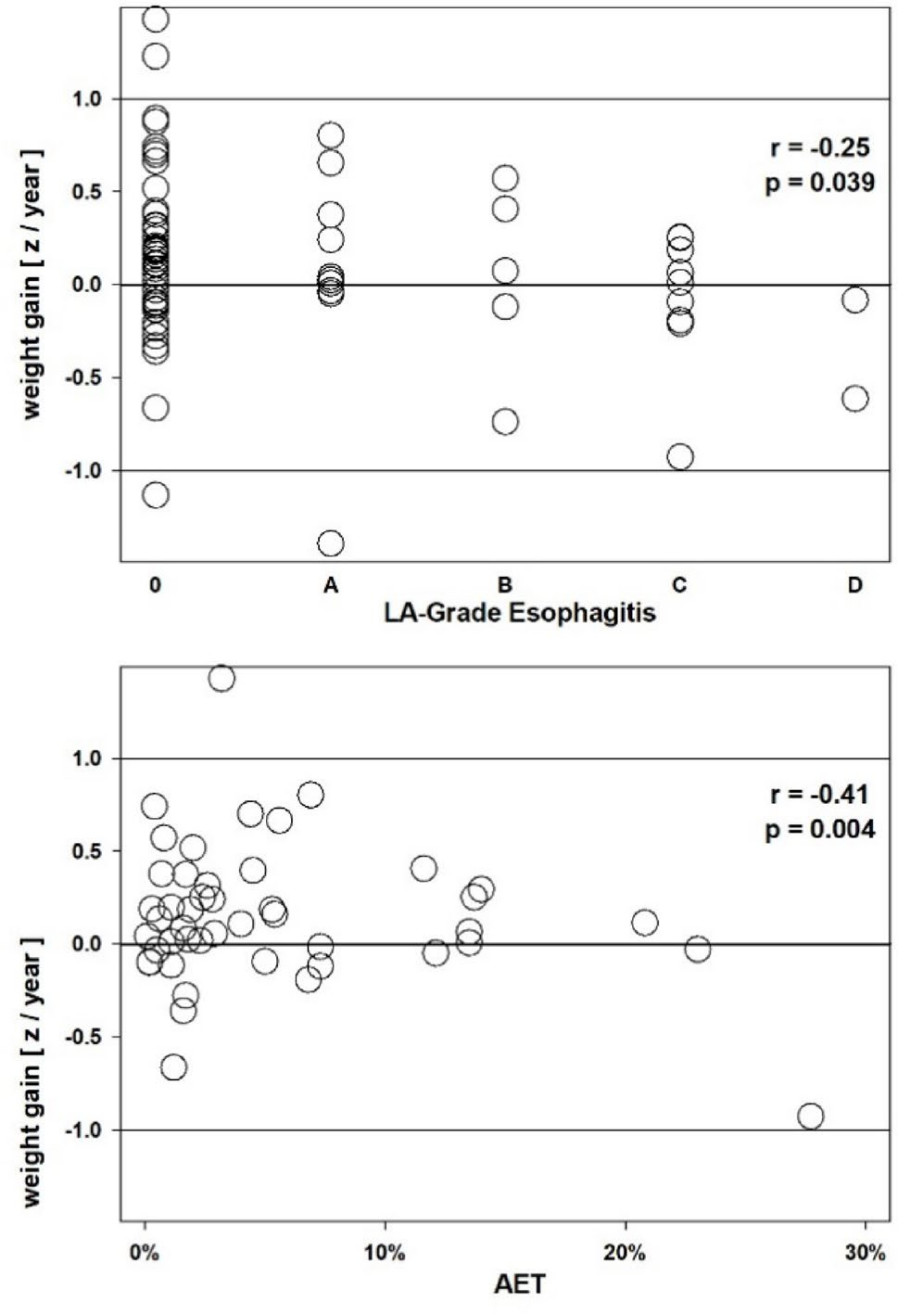
Negative correlation between esophagitis and reflux index with weight gain. Scatter plots show impaired thriving in CDH-patients by increasing grade of esophagitis and acid exposure time. *z* Standard Deviation from normal collective. *LA* Los Angeles, *AET* Acid Exposure Time

Reflux index (ρ = 0.34; *p* = 0.017) as well as reflux frequency (ρ = 0.43; *p* = 0.004) had a significant positive correlation with increasing grades of esophagitis. The reflux index (ρ = −0.41; *p* = 0.004) as well as esophagitis according to LA-classification (ρ = −0.25; *p* = 0.039) revealed a significant, negative correlation with weight gain (Fig. 3). This was not reflected by the results of the contrast studies. In children with reflux classified as pathologic, weight gain was lower, but the difference was not statistically significant (0.30z ± 0.62z vs. 0.17z ± 0.72z; *p* = 0.23).

### GER and pulmonary function

For obstructive pulmonary function (%FEV1/FVC), no significant associations with findings of reflux were detected in this cohort (AET *p* = 0.18, endoscopic esophagitis *p* = 0.52, histologic esophagitis *p* = 0.08, contrast study *p* = 1.0). However, in the subgroup of children with endoscopic esophagitis, a significant correlation between obstructive lung function and the endoscopic grade of esophagitis was observed (Los Angeles Classification) (ρ = −0.52, *p* = 0.046). The present data did not show any significant association between restrictive pulmonary function (%FVC) and reflux findings either (AET *p* = 0.45, endoscopic esophagitis *p* = 0.18, histologic esophagitis *p* = 0.63, contrast study *p* = 0.89). As %FVC was significantly lower (*p* = 0.014) in patients with large defects, i.e., C and D (68.1%±13.3%) compared to A and B (92.4%±28.1%), the interaction between defect size and %FVC was included into the ANCOVA. This statistical model could not detect any significant association between ventilatory restriction and the parameters of reflux findings.

### Predisposing factors for pathologic findings related to GER/GERD

A multinomial logistic regression model was performed with prematurity, laterality, defect size, liver herniation, patch use, operative approach and ECMO-therapy as potential predisposing factors. The only two significant predictors were defect size which had a significant, positive regression coefficient when running the model for acid exposure time (ß = 1.99 ± 0.54, *p*<0.001) and for endoscopic esophagitis (ß = 0.91 ± 0.44, *p* = 0.038) as well as liver-up with a significant positive regression coefficient for reflux count (2.40 ± 0.81, *p*=0.003) (Table 2).

**Table 2:** Defect size and liver-up as risk factors of esophagitis and pathologic pH-MII

	Potential Predisposition, Estimates of Regression Coefficients ± Standard Error							
	Prematurity	Laterality	Defect Size	Liver Pos.	Patch	Cone-Patch	Laparotomy vs. thoracoscopy	ECMO vs. no ECMO
	< 37wg	left vs. right	A, B, C, D (CDH-SG)	liver-up vs. down	any patch vs. no patch	cone patch vs. no cone		
Sample size (total 964)	195 (md = 37)	798 vs. 166 (md = 0)	86, 250, 249, 62 (u* = 317)	420 vs. 355 (md = 189)	662 vs. 246 (both = 4, md = 52)	562 vs. 100	698 vs. 158 (converted = 51, md = 57)	330 vs. 633 (md = 1)
AET	−0.22 ± 0.73	2.11 ± 2.75	1.99 ± 0.54	0.12 ± 0.75	12.41 ± 233	−15.08 ± 233	−2.01 ± 2.52	−0.87 ± 0.65
	*0.768*	*0.443*	***< 0.001***	*0.877*	*0.958*	*0.948*	*0.425*	*0.178*
Reflux Count	−0.02 ± 0.79	−11.77 ± 388	0.79 ± 0.51	2.40 ± 0.81	13.15 ± 229	−14.50 ± 229	−0.50 ± 2.45	−0.86 ± 0.70
	*0.983*	*0.976*	*0.117*	***0.003***	*0.954*	*0.949*	*0.839*	*0.220*
Endosc. Esophagitis	0.59 ± 0.63	1.09 ± 1.28	0.91 ± 0.44	−0.24 ± 0.91	11.87 ± 297	−11.37 ± 297	−0.12 ± 1.56	0.03 ± 0.66
	*0.346*	*0.397*	***0.038***	*0.791*	*0.968*	*0.969*	*0.940*	*0.964*
Histol. Esophagitis	0.37 ± 0.64	10.91 ± 189	0.55 ± 0.61	−0.76 ± 0.98	11.84 ± 354	−12.38 ± 354	2.10 ± 1.54	0.49 ± 0.60
	*0.564*	*0.954*	*0.374*	*0.440*	*0.973*	*0.972*	*0.172*	*0.413*
Contrast Study	−0.41 ± 0.73		−0.28 ± 0.52	−0.16 ± 1.06	0.18 ± 3.42	−2.37 ± 2.44		0.67 ± 0.76
	*0.320*		*0.599*	*0.880*	*0.957*	*0.331*		*0.771*

Estimates of regression coefficients ± standard error are shown for the respective potential explanatory variables (column titles). Note, defect size and liver-up were the only variables that showed a significant positive relation with esophagitis and pathologic pH-MII. Significance levels are tabled in italic font, values < 0.05 are marked in bold font

*AET* acid exposure time, *Liver Pos.* Liver Position, u* unclassified as grading was first published in 2013 [12], *wg* gestational age in weeks

The laterality of the CDH had no significant impact in the regression model. (Twelve patients with bilateral CDH were calculated as left sided. 209/916 CDH were right sided.) Patch usage did not show any significance in the regression analysis for pathologic findings related to GERD either. Comparing different types of defect closure did not show any significant differences for the risk of pathologic findings between primary, patch or cone-patch closure in an ANOVA. Testing reflux in the contrast study as a dependent variable did not reveal any significant predictors.

## Discussion

In this single-center cohort of 916 congenital diaphragmatic hernia (CDH) survivors clinically relevant reflux was significantly associated with impaired weight gain and, in a subset of patients, with obstructive pulmonary dysfunction. Reflux-related symptoms showed only weak concordance with objective findings, underscoring the limited reliability of symptom-based screening. Together, these data support the need for structured, objective reflux surveillance, particularly in high-risk patients.

After a follow-up of 8.7 years GERD was objectified by EGD with optional pH-MII in 5.2% of the CDH survivors, which is lower than prevalence values of 10–16% reported by other centers [20, 21]. Accepting further diagnoses by lone-standing contrast studies (8.2%), the overall rate reaches the same range. The persistence of troublesome reflux into school age is consistent with earlier findings: Koivusalo et al. [10] reported increasing prevalence up to 55% at 10 years, while Yamoto et al. [33] demonstrated a gradual decline in the need for medical therapy over time, confirming the chronic but evolving nature of GERD in CDH survivors.

Importantly, no significant association between endoscopic and histologic findings and reported symptoms as well as a poor association between pH-MII findings and symptoms suggests that symptom inquiry has low predictive value for identifying CDH-patients at risk for GER-related pathologies. Reportedly, erosive esophagitis can present without symptoms [4]. Similarly, the I-GERQ-R questionnaire has low accuracy compared with pH-impedance findings, and symptoms do not correlate with acid exposure or histologic esophagitis [23, 24, 27, 28].

No cases of Barrett’s esophagus or malignancy were identified in the present cohort to date. However, Pulvirenti et al. reported a relatively high prevalence of Barrett mucosa (2 out of 30 cases) in adolescents and young adults with a history of CDH [21].

Given the unreliability of symptom inquiry, the high prevalence of GER and the considerable risk of secondary morbidity, routine reflux diagnostics independent of reported symptoms have been integrated into the structured adolescent follow-up program at Mannheim University Medical Center since 2022. This strategy should be understood as a CDH-specific, risk-adapted approach. The comparatively high prevalence of GERD reported by other centers in smaller but intensively investigated CDH cohorts may suggest that reflux-related sequelae might be underdiagnosed. Of particular concern is the repeatedly described occurrence of Barrett’s esophagus in adolescents and young adults with CDH. Along similar lines, other authors have advocated routine endoscopic follow-up in CDH survivors to enable early detection and treatment of GER-related pathologies [10, 18, 35]. Within this specific high-risk context, endoscopic assessment may therefore serve as a targeted surveillance tool to prevent long-term complications in a vulnerable population. Here, it has to be considered that early-life screening and treatment do not necessarily prevent later GERD [20] as the age distribution of reflux in the present cohort confirms. To date, no unified national or international consensus exists on the timing of reflux screening in CDH patients. It has been suggested to adopt surveillance strategies from patients with esophageal atresia [35].

Patients with large diaphragmatic defects and liver-up position were more frequently affected by pathological reflux, which is consistent with previously published data [14, 16, 17] supporting consideration of a risk-stratified surveillance concept. The use of a patch might help to mitigate tension on the diaphragmatic hiatus [31]. In line with this, it did not appear as an independent risk factor.

Beyond mucosal changes, further sequelae of GERD could be demonstrated in this cohort: Impaired thriving was significantly associated with endoscopic esophagitis and AET. Thus, GERD should be considered a potentially modifiable factor in impaired thriving with other contributors such as increased respiratory effort and gastrointestinal dysmotility.

Furthermore, obstructive pulmonary function correlated with the severity of esophagitis consistent with the hypothesis of airway remodeling through chronic microaspiration. Patients with larger diaphragmatic defects exhibit greater pulmonary impairment, which may confound the relationship between reflux and lung function [3]. In line with this, forced vital capacity (FVC) was significantly lower in patients with large defects in the present study, reflecting the expected impact of impaired pulmonary development [2, 12]. Nevertheless, even after adjustment for defect size and liver position, no independent association between reflux parameters and obstructive or restrictive pulmonary function could be demonstrated.

The overall rate of ARS in this cohort (6.9%) is lower than in many published series, in which ARS is performed more liberally or even prophylactically [17]. Given the substantial complication rates and uncertain long-term efficacy, a restrictive, indication-based approach appears justified. Reported perioperative complication rates range from 12% to 42% in mixed congenital malformation cohorts [13, 19]. Long-term symptoms remain frequent, with moderate to severe reflux in 26% and dysphagia in 13% of patients at follow-up [26]. There is a broad consensus against routine prophylactic ARS in CDH patients [15, 17, 30, 31].

Still, most of the anti-reflux procedures in this cohort were performed within the first two years of life. In most of these infants, contrast studies constituted the primary—and frequently the sole—diagnostic modality guiding the decision for surgery. Historically, this approach aimed to objectify gastroesophageal reflux without exposing infants to anesthesia for esophagogastroduodenoscopy (EGD) or the discomfort of pH-MII. Similar strategies have been reported by other centers [7, 30]. In daily practice, pronounced reflux observed during contrast examination was often perceived as compelling and pathologic on an individual basis, particularly in symptomatic infants, and therefore carried considerable weight in surgical decision-making. Contrast studies in this cohort were not used as routine screening but were performed in symptomatic children. However, infants with reflux detected solely on contrast imaging exhibited only minimal differences in weight gain compared to those without radiologic reflux, and contrast findings alone did not translate into measurable clinical disadvantage. This discrepancy between individually convincing radiologic impressions and the lack of supportive outcome data raises concern that reliance on contrast-driven decision-making may—despite the low overall rate of ARS in this cohort—have contributed to surgical overtreatment in infancy. In line with current guideline recommendations favoring objective functional assessment, the present findings support a more standardized and evidence-based diagnostic approach before considering ARS in this vulnerable population.

This study has several important limitations. First, although data collection was prospective within a structured follow-up program, objective reflux diagnostics were not performed routinely in all patients at all time points but rather based on clinical indication and evolving protocols. Consequently, asymptomatic cases of GERD may have been missed, potentially leading to an underestimation of true prevalence. Second, no validated symptom questionnaire, such as the I-GERQ-R, was systematically applied. Finally, the long follow-up period was associated with progressive loss to follow-up, introducing the possibility of selection bias toward more symptomatic or health-conscious patients in later years.

## Conclusion

Gastroesophageal reflux is a common comorbidity in CDH and is associated with esophagitis, impaired thriving and possibly impaired lung function. These associations were reflected by esophagogastroduodenoscopy and impedance pH-metry, but not by contrast radiography. Structured follow-up with particular attention to gastroesophageal reflux is recommended. In selected high-risk patients with congenital diaphragmatic hernia - such as those with an initial defect size > C - EGD, with optional pH-MII monitoring, may be considered within a risk-adapted surveillance strategy. Contrast studies alone should not guide therapeutic decisions.

## Supplementary Information

Below is the link to the electronic supplementary material.

Supplementary Material 1

Supplementary Material 2

## Data Availability

The data supporting the findings of this study are not publicly available due to their sensitive nature, arising from the rarity of the congenital condition and the associated risk of patient identification, as assessed by the ethics committee. The data are held in controlled-access storage at the University Medical Center Mannheim and may be obtained from the corresponding author upon reasonable request.

## Abbreviations

AET: Acid exposure time
ANCOVA: Analysis of covariance
ANOVA: Analysis of variance
ARS: Anti–reflux surgery
ATS: American Thoracic Society
CDH: Congenital diaphragmatic hernia
CDH: SG–Congenital Diaphragmatic Hernia Study Group
EA: Esophageal atresia
ECMO: Extracorporeal membrane oxygenation
EGD: Esophagogastroduodenoscopy
ERS: European Respiratory Society
FEV1: Forced expiratory volume in 1s
FVC: Forced vital capacity
FEV1/FVC: FEV1/FVC ratio
GER: Gastroesophageal reflux
GERD: Gastroesophageal reflux disease
IGERQ–R: Infant Gastroesophageal Reflux Questionnaire–Revised
IQR: Interquartile range
LA: Los Angeles (classification of esophagitis)
LES: Lower esophageal sphincter
MII: Multichannel intraluminal impedance
PPI: Proton pump inhibitor
pH: MII–24–hour pH–metry with multichannel intraluminal impedance
SAS: Statistical Analysis System
SD: Standard deviation
U: Test–Mann–Whitney–U–Test
WG: Weeks of gestation
