# Supplementary material for: Gastroesophageal reflux in congenital diaphragmatic hernia survivors: objective diagnostics reveal clinically relevant disease beyond reported symptoms
Source: Pediatr Surg Int. 2026 May 19;42(1):235. doi: 10.1007/s00383-026-06409-3 (PMC13186794; doi:10.1007/s00383-026-06409-3)
Supplement: Supplementary file 1 — Supplementary Material 1 [file 383_2026_6409_MOESM1_ESM.docx]

|  | **Survivors at discharge**  **(n = 964)** | **EGD**  **± pH-MII**  **(n = 85)** | **Contrast Study**  **(n = 115)** |
| --- | --- | --- | --- |
| **Male** | 565 (58.6%) | 54 (63.5%) | 65 (56.5%) |
| **Prematurity^a^** | 194 (20.1%) | 20 (23.5%) | 34 (29.6%) |
| missing data | 31 (3.2%) | 6 (7.1%) | 3 (2.6%) |
| **Side of hernia** |  |  |  |
| left | 794 (82.4%) | 79 (92.9%) | 98 (85.2%) |
| right | 166 (17.2%) | 4 (4.7%) | 15 (13.0%) |
| bilateral | 4 (0.4%) | 2 (2.4%) | 2 (1.7%) |
| **ECMO** | 330 (34.2%) | 38 (44.7%) | 62 (53.9%) |
| missing data | 4 (0.4%) | 0 | 0 |
| **Liver-up in left-sided CDH** | 605 (62.8%) | 59 (69.4%) | 79 (68.7%) |
| missing data | 190 (19.7%) | 10 (11.7%) | 20 (17.4%) |
| **Surgical access** |  |  |  |
| open | 698 (72.4%) | 68 (80.0%) | 101 (87.8%) |
| minimally invasive | 158 (16.4%) | 3 (3.5%) | 4 (3.5%) |
| converted | 57 (5.9%) | 7 (8.2%) | 5 (4.4%) |
| missing data | 51 (5.3%) | 7 (8.2%) | 5 (4.4%) |
| **Type of repair** |  |  |  |
| primary | 246 (25.5%) | 9 (10.6%) | 13 (11.3%) |
| patch (all) | 662 (68.7%) | 68 (80.0%) | 97 (84.4%) |
| cone patch | 565 (58.6%) | 64 (75.3%) | 91 (79.1%) |
| patch+primary^c^ | 4 (0.4%) | 2 (2.4%) | 1 (0.9%) |
| missing data | 52 (5.4%) | 6 (7.1%) | 4 (3.5%) |
| **Defect size^b^** |  |  |  |
| A | 86 (8.9%) | 1 (1.1%) | 1 (0.9%) |
| B | 250 (25.9%) | 11 (12.9%) | 15 (13.0%) |
| C | 249 (25.8%) | 34 (40.0%) | 38 (33.0%) |
| D | 62 (6.4%) | 11 (12.9%) | 23 (20.0%) |
| missing data | 317 (32.9%) | 28 (32.9%) | 38 (33.0%) |

***Supplement 1: Characteristics of the local cohort of patients with CDH.*** *Data are presented as n (%).^a^ <37 weeks of gestation, ^b^ defect size according to CDH Study group[12]; data available only from 2009, ^c^ in bilateral hernia*
