# Supplementary material for: Gastroesophageal reflux in congenital diaphragmatic hernia survivors: objective diagnostics reveal clinically relevant disease beyond reported symptoms
Source: Pediatr Surg Int. 2026 May 19;42(1):235. doi: 10.1007/s00383-026-06409-3 (PMC13186794; doi:10.1007/s00383-026-06409-3)
Supplement: Supplementary file 2 — Supplementary Material 2 [file 383_2026_6409_MOESM2_ESM.docx]

Supplementary Material for Pediatric Surgery International

Manuscript title: Gastroesophageal Reflux in Congenital Diaphragmatic Hernia Survivors: Objective Diagnostics Reveal Clinically Relevant Disease Despite Mild Symptoms

Julia Elrod M.D.1§, Katharina Klensch 1§, Rasul Khasanov M.D. 1, Meike Weis M.D. Ph.D.2, Michaela Klinke Petrowsky M.D.1, Caroline Riemer M.D.1, Michael Boettcher M.D. Ph.D.1, Richard Martel M.D.1

1 Department of Pediatric Surgery, University Medical Center Mannheim, Medical Faculty Mannheim, Heidelberg University, Germany

2 Department of Clinical Radiology and Nuclear Medicine, University Medical Center Mannheim, Heidelberg University

§ These authors contributed equally to the work

Corresponding author: Richard D. Martel, M.D. E-Mail: richard.martel@medma.uni-heidelberg.de

| **Age at evaluation** |  | 6.2y (0.26y–18.4y) |
| --- | --- | --- |
|  |  |  |
| **Endoscopic Esophagitis** ^a^  (85 examined) |  | 31 (36.5%) |
|  | A | 10 (11.8%) |
|  | B | 8 (9.4%) |
|  | C | 10 (11.8%) |
|  | D | 3 (3.5%) |
|  | negative | 50 (58.8 %) |
|  | missing data | 4 (4.7%) |
|  |  |  |
| **Histological** **Esophagitis**  (59 examined) |  | 25 (42.4%) |
|  | mild | 16 (27.1%) |
|  | florid | 6 (10.2%) |
|  | ulcerous | 3 (5.1%) |
|  | negative | 28 (47.5 %) |
|  | missing data | 6 (10.2%) |
|  |  |  |
| **Acid exposure time** (58 examined) |  |  |
|  | >0.06 | 15 (25.9%) |
|  | >0.07 | 13 (22.4%) |
|  | >0.1 | 11 (19%) |
|  | <0.06 | 36 (62.1%) |
|  | missing data | 7 (12.1%) |
|  |  |  |
| **Increased reflux count ^b^** (50 examined) |  | 16 (32%) |
|  | negative | 28 (56%) |
|  | missing data | 6 (12%) |
|  |  |  |
| **Contrast Study** (115 examined) | reflux | 88 (76.5%) |
|  | negative | 27 (23.5%) |
|  |  |  |

***Supplement 2: Pathologic reflux is reflected by different diagnostic modalities in 123 cases out of 165 symptomatic patients.***

*Data are given as number and percentage of total and respective grade of pathologic results within the modalities. All modalities show decreasing proportions of affected patients at higher grades of severity. No Barrett’s esophagus was identified. The sum of pathologic findings across the modalities exceeds the number of 123 pathologic cases (13.4%) due to intermodal confirmative results. a Esophagitis graded according to the Los Angeles Classification. b More than 80 reflux episodes per 24 hours, classified according to the Lyon Consensus 2.0 [8]. Abbreviations: EGD esophagogastroduodenoscopy; MII = multichannel intraluminal impedance.*
